# *Fzd3* Expression Within Inner Ear Afferent Neurons Is Necessary for Central Pathfinding

**DOI:** 10.3389/fnins.2021.779871

**Published:** 2022-01-27

**Authors:** Zachary A. Stoner, Elizabeth M. Ketchum, Sydney Sheltz-Kempf, Paige V. Blinkiewicz, Karen L. Elliott, Jeremy S. Duncan

**Affiliations:** ^1^Department of Biological Sciences, Western Michigan University, Kalamazoo, MI, United States; ^2^Department of Biology, University of Iowa, Iowa City, IA, United States; ^3^Department of Biomedical Sciences, Western Michigan University Homer Stryker MD School of Medicine, Kalamazoo, MI, United States

**Keywords:** inner ear afferent, spiral ganglion neurons (SGN), *Frizzled3*, neuronal projections, Wnt/PCP

## Abstract

During development the afferent neurons of the inner ear make precise wiring decisions in the hindbrain reflective of their topographic distribution in the periphery. This is critical for the formation of sensory maps capable of faithfully processing both auditory and vestibular input. Disorganized central projections of inner ear afferents in *Fzd3* null mice indicate Wnt/PCP signaling is involved in this process and ear transplantation in *Xenopus* indicates that *Fzd3* is necessary in the ear but not the hindbrain for proper afferent navigation. However, it remains unclear in which cell type of the inner ear *Fzd3* expression is influencing the guidance of inner ear afferents to their proper synaptic targets in the hindbrain. We utilized *Atoh1-cre* and *Neurod1-cre* mouse lines to conditionally knockout *Fzd3* within the mechanosensory hair cells of the organ of Corti and within the inner ear afferents, respectively. Following conditional deletion of *Fzd3* within the hair cells, the central topographic distribution of inner ear afferents was maintained with no gross morphological defects. In contrast, conditional deletion of *Fzd3* within inner ear afferents leads to central pathfinding defects of both cochlear and vestibular afferents. Here, we show that *Fzd3* is acting in a cell autonomous manner within inner ear afferents to regulate central pathfinding within the hindbrain.

## Introduction

External information is relayed to the brain through precisely organized neural circuits that allow for sensory perception of the immediate environment (Cang and Feldheim, 2013; Fritzsch et al., 2019). The development of this neural wiring involves the growth and navigation of axons to their proper synaptic targets, often over large distances. Navigating axons integrate attractive and repulsive guidance information from the environment for directed extension (Kolodkin and Tessier-Lavigne, 2011). This guidance information comes in the form of both secreted and extracellular matrix-associated molecular cues of varying spatial distribution. Interpretation of these signals by growing axons into an appropriate chemotropic response allows for navigation and the formation of functional synapses with their targets (Maler et al., 1974; Chilton, 2006; Chai et al., 2015; Russell and Bashaw, 2017). This synaptic connection between sensory neurons and their centrally located targets generates a sensory map capable of faithfully transmitting information from the afferent divisions of sensory organs to their respective nuclei (Fritzsch et al., 2019).

The afferent neurons of the inner ear make precise wiring decisions in the hindbrain reflective of the topographic distribution of hair cells in the periphery (Maklad and Fritzsch, 2003; Appler and Goodrich, 2011). These specific decisions are essential for assembling the neuronal circuits dedicated for processing auditory or vestibular input. Auditory and vestibular signals are relayed from the periphery to the hindbrain by spiral ganglion neurons (SGNs) and vestibular afferents, respectively. SGNs send peripheral projections into the cochlea, where they innervate tonotopically organized hair cells. Within the cochlea, frequencies are encoded across a gradient with higher frequencies detected by hair cells in the base and lower frequencies detected by hair cells in the apex (Warr and Guinan, 1979; Bourk et al., 1981; Luo et al., 2009; Meyer et al., 2009). SGNs send their central processes to the cochlear nucleus (CN) located within the hindbrain, maintaining their tonotopic organization. Upon entering the hindbrain, SGNs will bifurcate, sending descending processes toward the posteroventral cochlear nucleus (PVCN) and dorsal cochlear nucleus (DCN), as well as ascending processes toward the anteroventral cochlear nucleus (AVCN), where they synapse on second order neurons within these nuclei (Wickesberg and Oertel, 1988; Ryugo and Parks, 2003). Vestibular afferents innervate the various peripheral sensory organs of the vestibular system and send central projections to specific areas of the vestibular nucleus (VN) and cerebellum (Dickman and Fang, 1996; Maklad and Fritzsch, 2003). While vestibular afferents and SGNs project together toward the hindbrain as the vestibulocochlear nerve, within the nerve, these two populations of neurons remain segregated, maintaining topographic fidelity to their respective peripheral sensory organs (Fritzsch, 1981).

The mechanisms that guide inner ear afferents to their peripheral hair cell targets has been a long-standing question (Fekete and Campero, 2007; Coate and Kelley, 2013; Coate et al., 2015). However, relatively little is known about the molecular guidance associated with central pathfinding. Remarkably, across all vertebrate species, auditory and vestibular afferents target the same respective dorsoventral columns of the hindbrain, indicating that central pathfinding may be occurring through a set of conserved guidance mechanisms (Duncan and Fritzsch, 2012). In support of this, transplantation studies in *Xenopus* and chick have suggested that inner ear afferents respond to conserved guidance cues during central circuit assembly (Elliott et al., 2013, 2015; Elliott and Fritzsch, 2018; Gordy et al., 2018). Furthermore, mouse SGNs project to the CN even in the absence of a majority of their second order synaptic targets (Maricich et al., 2009; Elliott et al., 2017), implying that the target cells themselves are not necessary for proper guidance. Taken together, these studies indicate that inner ear afferents may be responding to conserved spatiotemporal gradients of diffusible guidance cues for proper central pathfinding. Wnt/PCP signaling in the CNS is conserved across vertebrates and may be a promising avenue (Goodrich, 2008). Two Wnt/PCP signaling components, *Prickle1* (Yang et al., 2017) and *Frizzled3* (*Fzd3*) (Wang et al., 2002; Duncan et al., 2019), have been shown to be involved in central circuit assembly. In *Prickle1* mutants, apical SGNs aberrantly cover a more expansive area of the CN and overlap with basal fibers (Yang et al., 2017). A similar phenotype is observed in *Fzd3* null mutants, in which apical SGNs project in a disorganized manner within the CN (Duncan et al., 2019). Additionally, in *Fzd3* null mutants, some vestibular afferents will project out of the VN and into the CN in mice or into the lateral line nucleus in frogs, indicating a reliance on *Fzd3* within vestibular afferents as well (Duncan et al., 2019). Transplantation of ears between control and *Fzd3* knockdown *Xenopus* embryos demonstrated that a loss of *Fzd3* within the hindbrain did not result in aberrant vestibular afferent projections, whereas afferent projections were aberrant when *Fzd3* expression was downregulated within the inner ear (Duncan et al., 2019). This suggests that it is *Fzd3* expression within the ear and not in the hindbrain that is necessary for central guidance of auditory and vestibular afferents. *Fzd3* is expressed throughout the inner ear, including inner ear afferents (Lu et al., 2011; Ghimire et al., 2018) and hair cells. Within the inner ear afferents, *Fzd3* is expressed in a gradient, with a higher expression in the apex compared to the base (Duncan et al., 2019). This distinctive expression pattern suggests that the differential level of *Fzd3* expression may contribute to the proper segregation of SGNs to their respective regions of the CN; however, it remains unclear if *Fzd3* is acting in a cell autonomous manner within inner ear afferents or if *Fzd3* expression within their peripheral hair cell targets is the root of the central defects observed in mutants (Duncan et al., 2019). Either scenario seems likely as *Fzd3* has been shown to have a cell autonomous role in some instances (Wang et al., 2002; Chai et al., 2014), while in others, it is the expression of *Fzd3* within other cell types that is critical for proper axon pathfinding (Qu et al., 2014).

In the present work, we utilized two *Fzd3* conditional knockout models to selectively eliminate *Fzd3* from individual cell populations within the inner ear. We used *Neurod1-cre* (Li et al., 2012) to recombine floxed *Fzd3* (Hua et al., 2013) within inner ear afferents and *Atoh1-cre* (Matei et al., 2005) to recombine *Fzd3* within inner ear hair cells (Wang et al., 2005; Fujiyama et al., 2009; Yang et al., 2010). Our results show that *Fzd3*-mediated regulation of inner ear afferent central pathfinding relies on the expression of *Fzd3* within inner ear afferents.

## Materials and Methods

### Ethics Statement

All animal protocols used in these studies were approved by the Institutional Animal Care and Use Committee at Western Michigan University (20-11-01).

### Mice

*Atoh1-cre:Fzd3^f/f^* mice were produced by breeding *Atoh1-cre:Fzd3^f/+^* males with *Fzd3^f/f^* or *Fzd3^f/+^* females (Matei et al., 2005; Hua et al., 2013). *Neurod1-cre:Fzd3^f/f^* mice were produced by breeding *Neurod1-cre:Fzd^f/+^* males with *Fzd3^f/f^* or *Fzd3^f/+^* females (Li et al., 2012; Hua et al., 2013). All experiments were performed at postnatal day 0 (P0). Neonates were anesthetized by intraperitoneal injection of a lethal dose of Avertin (1.25% of 2.2.2-tribromoethanol) at a dose of 480 mg/kg and then perfused with 4% PFA using a peristaltic pump. Samples were stored in 4% PFA at 4°C until time of experiment. At least three biological replicates were used for each genotype in all experiments.

### Immunohistochemistry

To characterize the specific cell types in which Cre recombination would occur in both the *Atoh1-cre* and *Neurod1-cre* transgenic mouse lines, immunohistochemistry was performed on the cochleae of P0 mice. After fixation, ears were dissected to expose the cochleae. Samples were left in 1X PBS for 1 h. Samples were then washed in 0.05% Tween-20/PBS. Blocking was performed using 5% donkey serum, 0.5% Triton-X, and 1% bovine serum albumin in 1X PBS at room temperature on a rocker. After a quick rinse using 0.05% Tween-20/PBS, primary antibodies were added to the samples. Primary antibodies include Myo7a rabbit (1:500, Catalog #25-6790, Proteus Biosciences, Inc.) and Neurofilament Heavy Chain 200 kDa (NF200) chick (1:200, Catalog ID NFH, Aves), diluted in blocking solution. Samples were left in primary antibody solution at 4°C on a rocker for 2 days. Samples were then rinsed four times with 0.05% Tween-20/PBS, with each rinse being 30 min in duration. A fluorescent secondary (anti-rabbit, 1:1,000, Catalog #A31573 Invitrogen, or anti-chick, 1:1,000, Catalog #A11039, Invitrogen) was diluted in blocking solution and then added to each sample. Samples were left overnight at 4°C on a rocker. Samples were then washed three times with 0.05% Tween-20/PBS, with each wash being 30 min in duration. Samples were then mounted on glass slides using glycerol and imaged with a Nikon C2 Confocal Microscope.

### RNAscope

To confirm the loss of *Fzd3* in the conditional knockout mouse models, RNAscope^®^ was used to label individual *Fzd3* mRNA in the inner ears from P0 *Atoh1-cre*:*Fzd3^f/f^*, *Neurod1-cre*:*Fzd3^f/f^*, and respective control littermate mice. Three ears from each genotype were removed from the skull, post-fixed in 4% PFA for an hour, and stored in 70% EtOH until processing. Inner ears were then dissected in 70% EtOH to remove the cartilage around the entire cochlea and the ventral most part of the vestibular portion. Dissected inner ears were processed as previously described for whole-mount RNAscope^®^ (Kersigo et al., 2018). Ears were transferred to 70% MeOH, dehydrated to 100% MeOH, and rehydrated through a series of MeOH/0.1% Tween 20 in RNase-free phosphate-buffered saline (PBT) dilutions. Ears were treated with Protease III solution (Advanced Cell Diagnostics 322337, 1x) for 18 min and washed three times in PBT for 5 min each. Ears were incubated in probe solution for *Fzd3* (Advanced Cell Diagnostics, 404891, C1) and *Ubiquitin C* (Advanced Cell Diagnostics, 310771, C3) for ears from *Atoh1-cre*:*Fzd3^f/f^* and control littermates and in probe solution for *Fzd3* (Advanced Cell Diagnostics, 404891, C1) and *TrkC/Ntrk3* to label inner ear neurons (Fariñas et al., 2001; Green et al., 2012; Kersigo et al., 2018; Duncan et al., 2019) (Advanced Cell Diagnostics, 423621, C2) for ears from *Neurod1-cre*:*Fzd3^f/f^* and control littermates. Ears were incubated in their respective probe solutions overnight at 40°C with gentle agitation following manufacturer’s instructions. After overnight incubation, the probe solution was removed, and ears were washed in RNase-free 0.2x saline sodium citrate (SSC) three times for 15 min each. Ears were incubated in AMP1 solution (Advanced Cell Diagnostics, 1x) at 40°C for 35 min and washed three times in SSC. Ears were then incubated in AMP2 solution (Advanced Cell Diagnostics, 1x) at 40°C for 20 min and washed three times in SSC. Ears were incubated in AMP3 solution (Advanced Cell Diagnostics, 1x) at 40°C for 35 min and washed three times in SSC. Ears were incubated in AMP4 solution (Advanced Cell Diagnostics, Alt C, 1x) at 40°C for 20 min and washed three times in SSC.

Ears from *Atoh1-cre*:*Fzd3^f/f^* and control littermates were transferred to PBS, blocked for 1 h in 5% normal goat serum (NGS), and incubated with antibody against Myo7a (1:400; Proteus Biosciences, Inc.) in PBS overnight at 37°C to label hair cells. Ears were washed three times in PBS for 1 h each before blocking in 5% NGS/0.1% Triton X-100 in PBS. Ears were incubated in goat-anti-rabbit 647 secondary antibody (1:500; Alexa) and Hoechst stain (1:1,000) in PBS overnight at 4°C. Ears were washed three times in PBS for 1 h each. Ears from *Neurod1-cre*:*Fzd3^f/f^* and control littermates were incubated in a DAPI solution (Advanced Cell Diagnostics, 1x) at 4°C overnight and washed three times in PBS. All ears were mounted on a slide in Vectashield^®^ (Vector Laboratories, Inc.). Images were acquired using a Leica SP8 confocal microscope with LAS X software.

### Lipophilic Dye Tracing

To label specific inner ear afferents, tissue on the exterior of the skull was dissected to reveal the lateral half of the inner ear. For cochlear labeling, pieces of lipophilic dye-soaked filter paper were placed in the base of the cochlea (NeuroVue^®^ Red) and in the apex of the cochlea (NeuroVue^®^ Jade). For vestibular labeling, lipophilic dye-soaked filter paper was placed at the junction of the anterior cristae and utricle (NeuroVue^®^ Maroon) (Tonniges et al., 2010; Duncan et al., 2011). Placement was consistent for both control and mutant samples. Heads were then placed in vials containing 4% PFA and incubated at 37°C for 3 days to allow for dye diffusion. Following incubation, heads were hemisected and the brain was removed. The brain was flat-mounted with lateral side up on a slide in glycerol and imaged within 1 h of preparation. All imaging was performed using a Nikon Confocal C2 Microscope with NIS software.

### Afferent Reconstruction

To reconstruct individual afferents, Z-stack images were loaded into Fiji-ImageJ software. Ten individual apical afferents per sample were manually traced in the AVCN using the Simple Neurite Tracer plugin under the Segmentation tab by selecting points along a single fiber. Selected afferents were approximately 10 μm apart throughout the entire AVCN. The tracing started after the bifurcation of the neurite. If an axon could not be traced at the exact 10 μm distance, the next closest axon was selected. Images were imported into CorelDraw software and individual tracings were relabeled with different colors denoting their position within the AVCN. Neurons from three mutant and three littermate control mice were analyzed.

### Quantification of Axon Crossings

The reconstructed axons were examined for the number of times that an axon crossed another axon when projecting. The number of times that axons crossed one another was averaged and standard deviation was calculated in both control and mutant samples. A Student’s *t*-test was performed to assess significance of the data. Significance was determined at *p* < 0.05.

## Results

### Central Tonotopic Organization of Spiral Ganglion Neurons Is Unaffected by Loss of *Fzd3* Within Peripheral Hair Cells

**FIGURE 1:**
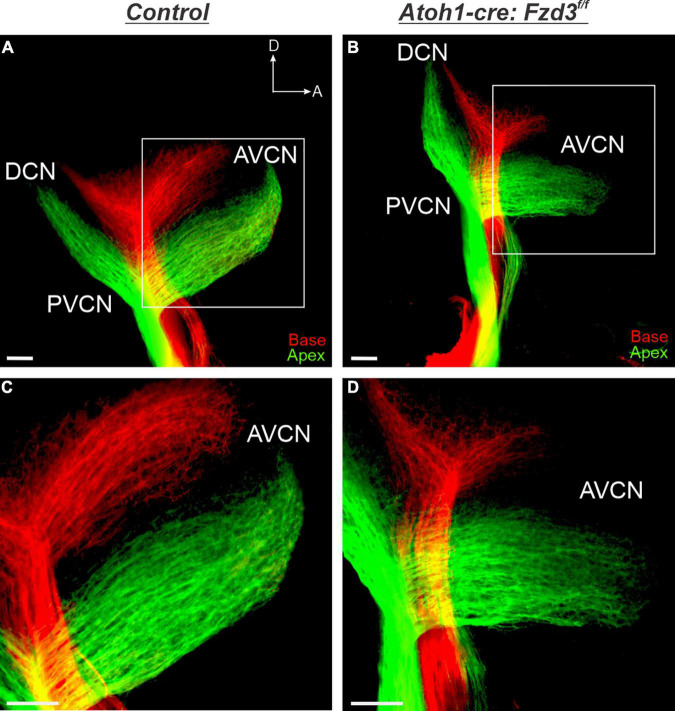
Cochlear afferent central pathfinding unaffected by loss of *Fzd3* within hair cells. Lipophilic dyes were applied to the base (red) and apex (green) of control **(A,C)** and *Atoh1-cre:Fzd3^f/f^* mutant **(B,D)** cochleae at P0 and their central projections were analyzed **(A–D)**. In the control, afferent neurites from the base and apex remain segregated and bifurcate sending projections toward the DCN and AVCN **(A,C)**. In the *Atoh1-cre:Fzd3^f/f^* mutant, afferent neurites from the base and apex also remain segregated and bifurcate sending projections toward the DCN and AVCN **(B,D)**. Higher magnification of the AVCN **(C,D)** showing the segregation of basal and apical afferents in both control **(C)** and *Atoh1-cre:Fzd3^f/f^* mutant **(D)** central projections. CN, cochlear nucleus; AVCN, anteroventral cochlear nucleus; DCN, dorsal cochlear nucleus; PVCN, posteroventral cochlear nucleus; A, anterior; D, dorsal. Orientation for all panels the same as **(A)**. Scale bars, 100 μm. Three mutant animals and three littermate controls were analyzed.

Since *Fzd3* has been shown to be expressed within the cochlear hair cells (Wang et al., 2006; Stuebner et al., 2010; Qu et al., 2014; Duncan et al., 2019), we assessed whether loss of *Fzd3* in this cellular population affected central pathfinding of SGNs through a non-cell autonomous manner. The basic helix-loop-helix (bHLH) transcription factor *Atoh1* is expressed in hair cells and has been shown to play a critical role in their fate determination and differentiation (Bermingham et al., 1999). Thus, we used an *Atoh1-cre* line that has been shown by several studies to drive *Cre* expression within inner ear hair cells (Supplementary Figure 1; Matei et al., 2005; Perrin et al., 2010, 2013; Ueyama et al., 2014; Tarchini et al., 2016; Kannan-Sundhari et al., 2020) to conditionally delete *Fzd3* (Supplementary Figure 1). Although we found variability in *Atoh1-cre* driving tdTomato expression in supporting cells, all samples expressed tdTomato within hair cells and there was no evidence of tdTomato in inner ear afferents. Application of lipophilic dyes into the base (red) and apex (green) of control cochleae showed that SGNs project in an organized manner toward and within the CN, with basal and apical fibers remaining segregated based on their tonotopic distribution in the periphery (Figures 1A,C), consistent with previous morphological reports (Wickesberg and Oertel, 1988; Ryugo and Parks, 2003). Similarly in *Atoh1-cre:Fzd3^f/f^* mutants, tonotopic organization is maintained with basal fibers projecting more dorsally than apical fibers (Figures 1B,D). Overall, there appears to be no difference in tonotopic distribution of SGNs within the CN between control (Figures 1A,B) and *Atoh1-cre:Fzd3^f/f^* mutants (Figures 1B,D). This indicates that *Fzd3* expression within hair cells is not necessary for proper central pathfinding of SGNs.

**FIGURE 2:**
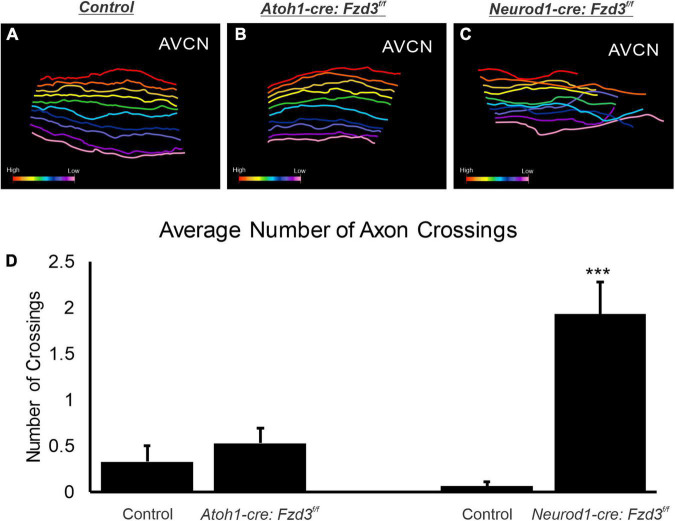
Analysis of cochlear afferent central projections following conditional deletion of *Fzd3*. Reconstruction of 10 evenly spaced apical afferents in the AVCN of control **(A)**, *Atoh1-cre:Fzd3^f/f^* mutant **(B)**, and *Neurod1-cre:Fzd3^f/f^* mutant **(C)**. Colored gradient bar represents the transition from higher frequency reconstructed fibers (red) to lower frequency reconstructed fibers (pink) **(A–C)**. The mean number of times an axon crossed another axon in littermate control and mutant samples **(D)**. Error bars represent standard error of the mean. ***p < 0.001.

To confirm that there was no difference between control and *Atoh1-cre:Fzd3^f/f^* mice central projections, we investigated the trajectories of afferents as they projected within the AVCN. To this end, ten apical SGNs were traced per animal for each of three control and three *Atoh1-cre:Fzd3^f/f^* mutants. In both control and *Atoh1-cre:Fzd3^f/f^* mice, adjacent fibers preserved relative separation between each other with infrequent path crossing events (Figures 2A,B). The number of times that individual axons crossed one another within the AVCN did not statistically differ between control and *Atoh1-cre:Fzd3^f/f^* mutants (Student’s *t*-test, *p* > 0.05) (Figure 2D). These findings indicate that *Fzd3* expression within inner ear hair cells does not influence the tonotopic distribution of SGNs within the hindbrain.

### Loss of *Fzd3* in Spiral Ganglion Neurons Results in Aberrant Tonotopic Organization Within the Cochlear Nucleus

**FIGURE 3:**
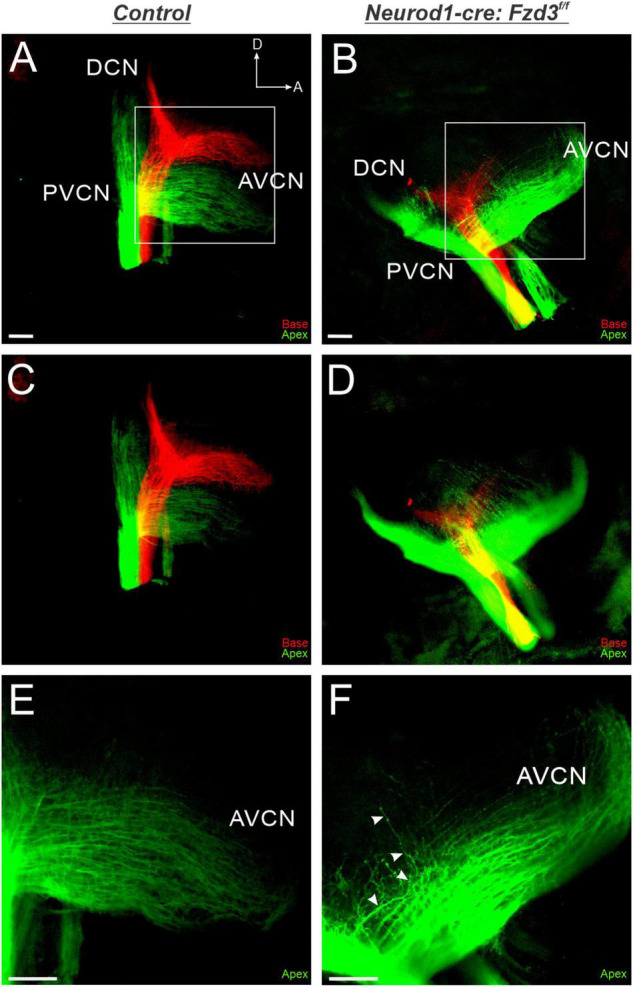
Loss of *Fzd3* in cochlear afferents results in central pathfinding defects. Lipophilic dyes were applied to the base (red) and apex (green) of control **(A,C,E)** and *Neurod1-cre:Fzd3^f/f^* mutant **(B,D,F)** cochleae at P0 and their central projections were analyzed **(A–F)**. In the control, afferent neurites from the base and apex remain segregated and bifurcate, sending projections toward the DCN and AVCN **(A,C)**. In contrast some apical afferents in *Neurod1-cre:Fzd3^f/f^* mutants project more dorsally, resulting in a loss of segregation between basal and apical fibers **(B,D)**. Stack images rendered at different depths in the CN for control **(C)** and *Neurod1-cre:Fzd3^f/f^* mutant **(D)** central projections. Higher magnification of the AVCN **(E,F)** showing apical afferents projecting parallel to each other in the control **(D)**. Some apical afferents in the *Neurod1-cre:Fzd3^f/f^* mutant (F) do not maintain separation from other fibers as they project toward the AVCN resulting in frequent crossover events. The white arrows show the trajectory of a single fiber as it projects in the AVCN of the *Neurod1-cre:Fzd3^f/f^* mutant **(F)**. CN, cochlear nucleus; AVCN, anteroventral cochlear nucleus; DCN, dorsal cochlear nucleus; PVCN, posteroventral cochlear nucleus; A, anterior; D, dorsal. Orientation for all panels the same as **(A)**. Scale bars, 100 μm. Three mutant animals and three littermate controls were analyzed.

Since *Fzd3* has also been shown to be expressed in cochlear afferents (Supplementary Figure 1; Lu et al., 2011; Ghimire et al., 2018; Duncan et al., 2019), we next investigated if expression specifically within SGNs was influencing proper central pathfinding within the CN. The proneural bHLH transcription factor *Neurod1* has been shown to play a role in inner ear afferents early during differentiation (Appler and Goodrich, 2011; Macova et al., 2019). We characterized a *Neurod1-cre* mouse line (Li et al., 2012) by combining it with a tdTomato reporter (*Neurod1-cre:tdTomato^+^*) to verify that *Cre* expression was specifically driven within inner ear afferent neurons (Supplementary Figure 1). *Neurod1* is expressed in SGNs beginning at E8.75 (Liu et al., 2000) and therefore eliminates *Fzd3* before inner ear afferents delaminate from the otocyst (Ruben, 1967; Kim et al., 2001). As before, control littermates showed the stereotypical projections of apical and basal SGNs as visualized by application of lipophilic dyes into the base (red) and apex (green) (Figures 3A,C). In contrast, in *Neurod1-cre:Fzd3^f/f^* mice, SGNs did not maintain the same degree of segregation between basal and apical fibers. Frequently, apical fibers would project dorsally into regions typically innervated by basal fibers. This dorsal projection of apical fibers occurred in both the AVCN and PVCN of *Neurod1-cre:Fzd^f/f^* mutant mice (Figures 3B,D). Even within the apical SGNs alone, many projections in the AVCN of *Neurod1-cre:Fzd3^f/f^* mutant mice did not maintain the same degree of tonotopic organization (Figure 3F) when compared to control mice (Figure 3E). To better visualize this divergence from controls, 10 apical SGNs were traced for both 3 control and 3 *Neurod1-cre:Fzd3^f/f^* mutants within the AVCN. In control mice, adjacent fibers maintained parallel separation between each other with infrequent path crossing events (Figure 2A). In contrast, apical fibers from *Neurod1-cre:Fzd3^f/f^* mice, frequently intersected with adjacent fibers as they projected (Figure 2C). The number of times that individual axons crossed one another within the AVCN was significantly greater in *Neurod1-cre:Fzd3^f/f^* mice (Figure 2D) than in controls (Figure 2D) (Student’s *t*-test, *p* < 0.001). Overall, these findings suggest that *Fzd3* is acting in a cell autonomous manner within SGNs to influence central pathfinding within the CN.

### Loss of *Fzd3* in Vestibular Afferents Results in Central Pathfinding Defects

**FIGURE 4:**
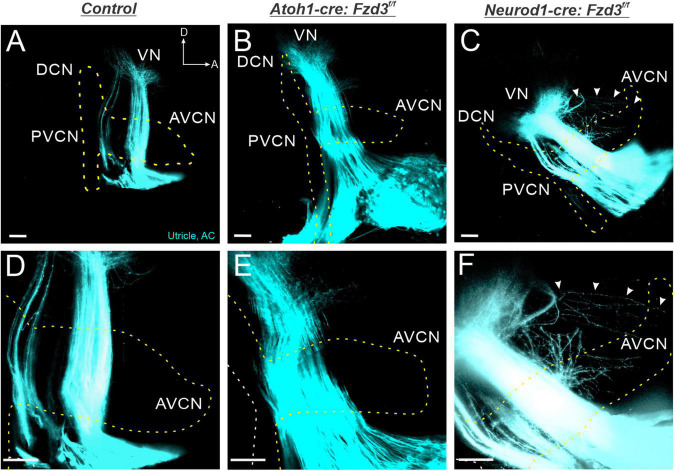
Vestibular afferents project out of the VN following the loss of *Fzd3*. Lipophilic dyes were applied to the apex (projection boundary outlined by dotted yellow line) and the utricle/anterior crista (cyan) of *control* **(A,D)**, *Atoh1-cre:Fzd3^f/f^* **(B,E)**, and *Neurod1-cre:Fzd3^f/f^* mutant **(C,F)** cochleae at P0 and their central projections were analyzed **(A–F)**. In the control, vestibular afferents project toward the VN and remain segregated from cochlear afferents **(A)**. In the *Atoh1-cre:Fzd3^f/f^* mutant, vestibular afferents also remain segregated from cochlear afferents and send projections toward the VN **(B)**. In contrast, vestibular afferents in the *Neurod1-cre:Fzd3^f/f^* mutant project out of the VN into the AVCN resulting in a loss of segregation between cochlear and vestibular afferents **(C)**. Higher magnification of the VN and AVCN **(D–F)** showing the projection of vestibular and apical afferents in the control **(D)**, *Atoh1-cre:Fzd3^f/f^* **(E)** and *Neurod1-cre:Fzd3^f/f^* mutant **(D)**. The white arrows show the projection of a single vestibular afferent out of the VN, into the AVCN of the *Neurod1-cre:Fzd3^f/f^* mutant **(C,F)**. AC, anterior cristae; VN, vestibular nucleus; AVCN, anteroventral cochlear nucleus; DCN, dorsal cochlear nucleus; PVCN, posteroventral cochlear nucleus; A, anterior; D, dorsal. Orientation for all panels the same as **(A)**. Scale bars, 100 μm. Three mutant animals and three littermate controls were analyzed.

Given that *Fzd3* is acting in a cell autonomous manner within SGNs for proper central pathfinding (Figures 1–3), we next investigated whether *Fzd3* plays a similar functional role within vestibular afferents. Since *Fzd3* is expressed in vestibular afferents (Lu et al., 2011; Ghimire et al., 2018) as well as their peripheral hair cell targets (Supplementary Figure 1; Wang et al., 2006), we evaluated in which of these cell types *Fzd3* expression contributes to proper vestibular afferent central pathfinding. To achieve this, we utilized *Atoh1-cre* and *Neurod1-cre* lines to conditionally delete *Fzd3* from the vestibular hair cells and the vestibular afferents, respectively. Application of lipophilic dyes into the utricle/anterior cristae (cyan) and apex (yellow outline) of control mice revealed normal projection of vestibular afferents toward the VN, with stereotyped segregation from SGNs (Figures 4A,D). Similarly, in *Atoh1-cre:Fzd3^f/f^* mutants, SGNs and vestibular afferents remain segregated (Figures 4B,E). In contrast, vestibular afferents in *Neurod1-cre:Fzd3^f/f^* mutants, often project in a disorganized manner to the VN and as well as to the AVCN (Figures 4C,F). In these mice, some fibers are observed projecting first to the VN and from there to the AVCN. Additionally, many fibers appear to exit the vestibular nerve prematurely and project into areas typically innervated by SGNs. Altogether, these findings suggest that *Fzd3* is acting in a cell autonomous manner within vestibular afferents as well to facilitate proper central pathfinding to the VN.

## Discussion

**FIGURE 5:**
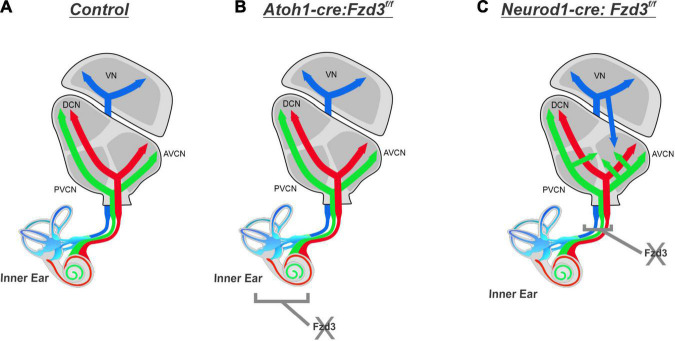
Proposed effect of *Fzd3* in inner ear afferent central pathfinding. Cochlear afferents from the base (red) and apex (green) project into the CN and bifurcate sending projections toward the DCN and AVCN while maintaining the tonotopic map established in the periphery **(A)**. Vestibular afferents (blue) project toward the VN and remain segregated from cochlear afferents **(A)**. Inner ear afferent central pathfinding is unaffected by loss of *Fzd3* within the hair cells in *Atoh1-cre:Fzd3^f/f^* mutants **(B)**. Loss of *Fzd3* within inner ear afferents in *Neurod1-cre:Fzd3^f/f^* mutants disrupts central pathfinding, resulting in aberrant projection of both apical SGNs and vestibular afferents **(C)**. VN, vestibular nucleus; CN, cochlear nucleus; AVCN, anteroventral cochlear nucleus; DCN, dorsal cochlear nucleus; PVCN, posteroventral cochlear nucleus.

The results presented here expand upon our previous study implicating a possible conserved role of *Fzd3* in inner ear afferent targeting of the correct dorsoventral column in the hindbrain (Duncan et al., 2019). Furthermore, using unique ear transplantation experiments in *Xenopus*, this previous study determined that *Fzd3* was dispensable within the hindbrain but was required by cells originating from the ear for proper afferent guidance centrally. Consequently, our present study was designed to determine which cell type(s) within the inner ear required *Fzd3* expression for appropriate targeting of inner ear afferent central projections. By utilizing conditional knockout mouse lines of *Fzd3* in hair cells (*Atoh1-cre*, Figures 1, 4) and in inner ear afferents themselves (*Neurod1-cre*, Figures 3, 4), we demonstrate that *Fzd3* acts in a cell autonomous manner within the inner ear afferents to influence proper central pathfinding of both SGNs and vestibular afferents (Figure 5). Conditionally knocking out *Fzd3* from the hair cells (Figures 1, 4) has no apparent effect on the central pathfinding of either inner ear afferent populations. Conditional deletion of *Fzd3* from the inner ear afferents leads to central pathfinding defects in both SGNs and vestibular afferents (Figures 3, 4), comparable to the central phenotype observed in *Fzd3* null mutants (Duncan et al., 2019). Consistent with the null mice, *Neurod1-cre:Fzd3^f/f^* mice display aberrant SGN pathfinding, especially by apical SGNs, as well as complete mistargeting of some vestibular afferents to the cochlear nucleus. We cannot rule out a minor non-autonomous effect from another cell type not examined in the current study.

Despite multiple studies looking into cell-specific requirements for *Fzd3* in axon guidance, variation exists between different cell types, with *Fzd3* having a cell autonomous role in some instances of neuronal circuit assembly but a non-cell autonomous role in others. While our data supports that *Fzd3* has a cell autonomous role in inner ear afferent central pathfinding, it is important to note that *Fzd3* does not always have a cell autonomous role in axon guidance within the central nervous system. A study examining the role of Wnt/PCP pathways in development of facial branchiomotor neurons suggested that *Fzd3* may have both cell autonomous and non-cell autonomous roles, but that they do not have to be considered separately (Vivancos et al., 2009). Their rationale, supported by the differing roles of *Fzd3* determined in other studies (Wang et al., 2002; Qu et al., 2010, 2014; Chai et al., 2014), proposes that *Fzd3* may function in the response of developing axons to chemoattraction cues (cell autonomous), but that *Fzd3* may act in surrounding cell types to influence axon guidance. The inner ear afferent neurons have been shown to have defects in their peripheral projections due to loss of *Fzd3* in a non-cell autonomous manner (Ghimire et al., 2018; Ghimire and Deans, 2019). Thus, the loss of *Fzd3* has a non-autonomous effect on the peripheral projections and our study shows an autonomous effect on the central projection of inner ear afferents. The cell autonomous nature of *Fzd3* in controlling the central pathfinding of inner ear afferent neurons is similar to that of 5-HT and mdDA neurons of the hindbrain (Fenstermaker et al., 2010), and commissural axons in the neural tube (Lyuksyutova et al., 2003; Onishi et al., 2013; Onishi and Zou, 2017). In both of these locations it is thought that Wnts are the ligand binding FZD3.

Some studies suggest that the role of *Fzd3*, at least in some instances, may be to establish pioneer neurons or to organize guidepost cells (Qin et al., 2020). Therefore, it may be the loss of these critical guides in absence of *Fzd3* that leads to disruption of normal projection patterns for developing axons. It is plausible that without *Fzd3*, an interaction that would normally occur between basal and apical fibers is missing, or a new interaction has been introduced, that is leading to the misprojections observed when *Fzd3* is conditionally deleted from inner ear afferent neurons. Conversely, there may be missing or newly present cues between SGN fibers and vestibular afferents, contributing to the presence of vestibular afferents in the CN. It is unclear if the defects seen by eliminating *Fzd3* using *Neurod1-Cre* is due to the loss from all neurons, or if the effects seen are due to elimination of *Fzd3* in subpopulations of neurons. This subpopulation effect of *Fzd3* will need to be clarified in future studies.

The graded expression of *Fzd3* across SGNs (Duncan et al., 2019) may explain why we observed greater central pathfinding defects in apical SGNs in the *Fzd3* mutants. Since *Fzd3* is expressed at higher levels in apical SGNs as compared to basal SGNs (Duncan et al., 2019), loss of *Fzd3* may have a more severe impact on the ability of apical afferents to properly navigate within the CN. This suggests that gradients of Wnt/PCP signaling components may be restricting the projection of a given SGN to a specific region of the CN, possibly with additional repulsive/attractive ques in the extracellular environment, as has previously been shown for the visual system. In the visual system, a gradient of another Wnt/PCP signaling component, *Ryk*, expressed across retinal ganglion cells, mediates repulsion from the graded expression of *Wnt3* ligand in the tectum. *Fzd* expression in the retinal ganglion cells mediates attraction that, together with *Ryk* repulsion, helps establish the dorsoventral retinal mapping to the tectum (Schmitt et al., 2006). Whether SGNs are directed to a particular dorsoventral region of the CN based on the FZD3 receptor concentration and the concentration of Wnt ligands released from the dorsal hindbrain remains to be explored. Furthermore, the extent that additional Wnt/PCP components, such as RYK or other FZD receptors, are involved in inner ear pathfinding is not yet known. *Fzd3* has been shown to work together with *Celsr3*, another PCP protein, in development of both the peripheral and central nervous system (Wang et al., 2002; Zhou et al., 2008; Vivancos et al., 2009; Qu et al., 2010, 2014; Chai et al., 2014). Wnt-mediated axon guidance has been well-established in other major neuronal tracts (Lyuksyutova et al., 2003; Liu et al., 2005; Keeble and Cooper, 2006; Fenstermaker et al., 2010) beyond the ear and eye and the expression of Wnts in the dorsal hindbrain is conserved across vertebrate species (Hernandez-Miranda et al., 2017; Elliott and Fritzsch, 2018). However, the issue of which Wnt ligand(s) play a role in central pathfinding of SGNs is complicated by the existence of 19 different Wnts in mammals that can bind to 10 different FZD receptors (Wang et al., 2012), as well as ROR (Minami et al., 2010) and RYK (Yoshikawa et al., 2003) receptors. Some promising candidates are *Wnt3a* and *Wnt5a*, based on their expression in hindbrain regions that the inner ear afferents are targeting (Louvi et al., 2007; Di Bonito and Studer, 2017) and their ability to bind to FZD3 (Dijksterhuis et al., 2014). *Wnt1* is also expressed in the same hindbrain region; however, it has not been shown to bind to FZD3 (Dijksterhuis et al., 2014). Identification of Wnt ligands/receptors involved in inner ear afferent central pathfinding will be critical in beginning to unravel the role Wnt/PCP signaling plays in this process.

## Data Availability Statement

The raw data supporting the conclusions of this article will be made available by the authors, without undue reservation.

## Ethics Statement

The animal study was reviewed and approved by the IACUC Western Michigan University.

## Author Contributions

ZS and EK carried out lipophilic dye and tdTomato experiments, data collection, and wrote the initial manuscript. KE carried out the RNAscope experiments. SS-K and PB aided in tissue collection and performed experiments. All authors conceived and designed the experiments, and edited the final manuscript.

## Conflict of Interest

The authors declare that the research was conducted in the absence of any commercial or financial relationships that could be construed as a potential conflict of interest.

## Publisher’s Note

All claims expressed in this article are solely those of the authors and do not necessarily represent those of their affiliated organizations, or those of the publisher, the editors and the reviewers. Any product that may be evaluated in this article, or claim that may be made by its manufacturer, is not guaranteed or endorsed by the publisher.
