# Supplementary figures and images for: Fzd3 Expression Within Inner Ear Afferent Neurons Is Necessary for Central Pathfinding
Source: Front Neurosci. 2022 Jan 27;15:779871. doi: 10.3389/fnins.2021.779871 (PMC8828977; doi:10.3389/fnins.2021.779871)

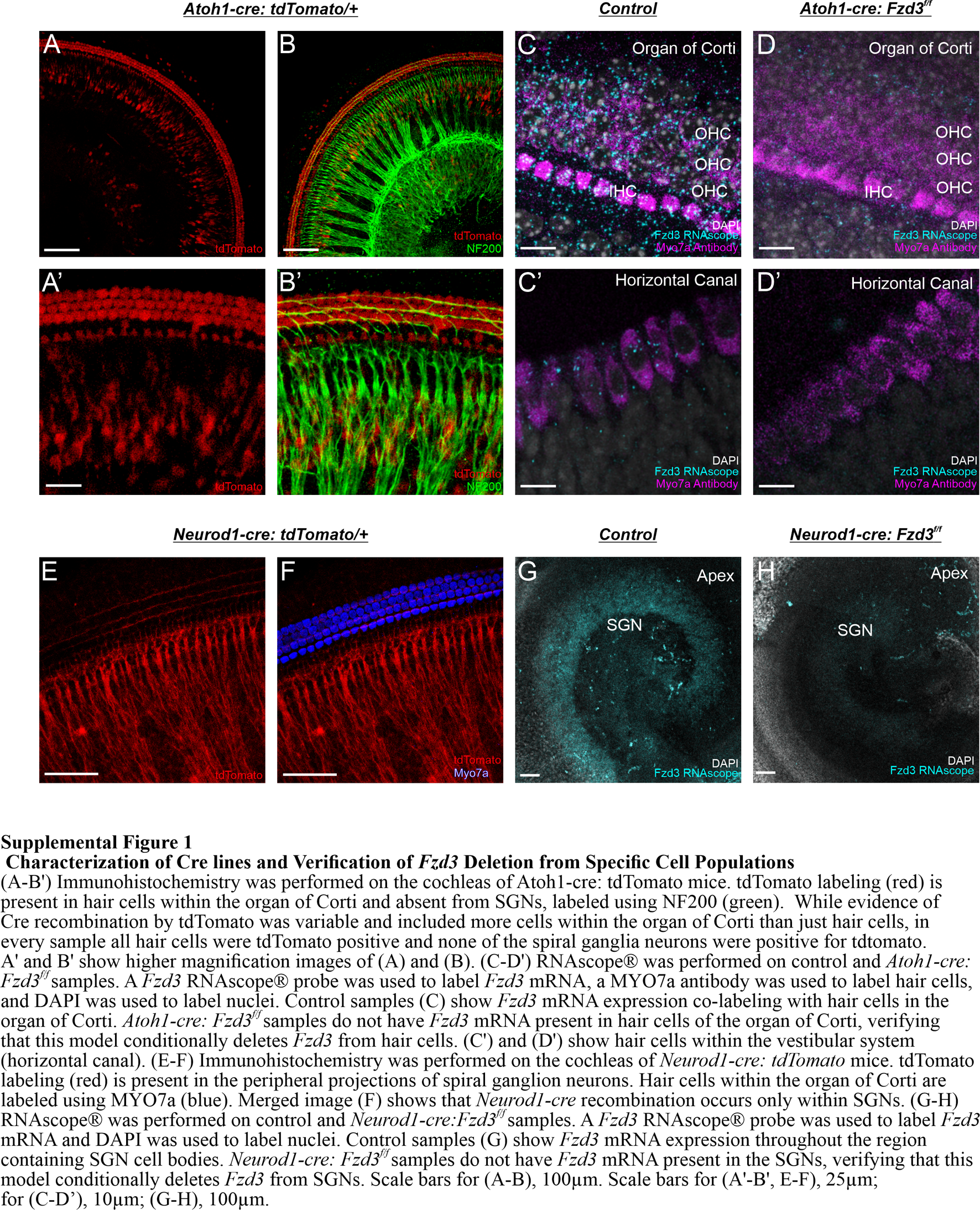

Supplement: Supplementary file 1 [file Image_1.tif]
